# Simple Strategies to Modulate the pH-Responsiveness of Lignosulfonate-Based Delivery Systems

**DOI:** 10.3390/ma15051857

**Published:** 2022-03-02

**Authors:** Massimo Sgarzi, Matteo Gigli, Charlotte Giuriato, Claudia Crestini

**Affiliations:** Department of Molecular Sciences and Nanosystems, Ca’ Foscari University of Venice, Via Torino 155, 30172 Venezia-Mestre, Italy; massimo.sgarzi@unive.it (M.S.); 839650@stud.unive.it (C.G.)

**Keywords:** lignin microcapsules, lignosulfonate, lignin fractionation, encapsulation of actives, ultrasonication, pH-responsive behavior, controlled release

## Abstract

The extensive use of non-degradable microplastics in a wide plethora of daily life products is causing serious pollution problems. More ecofriendly solutions are therefore urgently needed. In this context, the use of lignin, a largely available aromatic polymer, may represent a viable option. Due to the self-assembly ability of its molecules, lignin is in fact an ideal matrix for the fabrication of nanostructures. In this study, lignosulfonate microcapsules containing a limonene core were prepared and characterized in terms of their dimensions and of the physicochemical characteristics of the capsule-forming lignosulfonate molecules. The main purpose is to elucidate the key properties governing the pH-responsive behavior of the capsules to be able to achieve better control over the release kinetics of the entrapped compound(s). The results demonstrate that both the molecular weight and the concentration of sulfonate groups are the most important factors in this respect. Based on these findings, two strategies were followed to further tailor the capsules’ behavior: (i) fractionation of the starting lignosulfonate by solvent extraction and (ii) introduction of a specific additive in the formulation. The first approach permitted to fabricate highly resistant capsules both in acidic, as well as in alkaline conditions, while in the second case the chemical structure of the additive, the diester diveratryl sebacate, allowed for fast kinetics of release, as values above 70% were reached after 24 h of incubation at pH 4 and pH 12.

## 1. Introduction

Lignin is a widely available polymer (about 70 Mt of technical lignins are annually produced) [1] and, due to the aromatic structure rich in hydroxyl groups, it could be used as renewable starting material for the generation of a broad range of products [2,3,4,5]. Nevertheless, lignin is a complex material. Its chemical structure, which lacks a primary repeating unit, not only varies from species to species and even within the same plant, but is further altered by the processes used to separate lignin from cellulose and hemicellulose [6]. Specifically, the so-called technical lignins are byproducts of the pulp and paper and modern biorefinery industries, which adopt harsh conditions for lignin extraction, giving rise to highly modified and heterogeneous materials, whose structure significantly differs from that of native lignin and strictly depends on the extraction protocol [7]. For these reasons, coupled with the fact that depolymerization and chemical functionalization strategies yield complex mixtures which require long and costly procedures to achieve products with an acceptable degree of purity, lignin valorization is highly challenging. Hence, the use of technical lignins without any additional transformations for the synthesis of micro- and nanostructures, especially in the form of particles, capsules and fibers, is attracting considerable attention [6,8,9]. In the last years, a few works have been dealing with the generation of lignin-based microcapsules (LMCs) for the entrapment and targeted release of active compounds [10,11,12,13,14,15]. Lignins are indeed very suitable matrices for microencapsulation purposes as, together with intrinsic self-assembly features, they display highly peculiar characteristics of great interest for the protection of the core species, such as antimicrobial and antioxidant properties, as well as the ability to absorb UV-light [16,17,18,19]. Recently, our group mapped the stability of lignosulfonate-derived LMCs by varying a plethora of parameters such as temperature, pressure, pH, salinity and various solvents and surfactants demonstrating that the capsules disassemble at acidic pH values and high salinity [20].

By exploiting the self-assembly ability of lignosulfonate molecules, activated using ultrasounds, LMCs with limonene core were for the first time generated. The lignin ability to self-aggregate origins from the π–π stacking interactions of its aromatic groups and from the intermolecular hydrogen bonds. The hydrophobic core of lignosulfonate-based LMCs can host species poorly soluble in water (such as limonene or several other active ingredients), while the presence of sulfonate groups endows the system with water dispersibility [11,20].

Limonene is indeed a biobased widely available and non-toxic monocyclic monoterpene which can be used in a broad range of applications ranging from cosmetics to pharmaceuticals and agri-food industry, e.g., as substitute of traditional hazardous pesticides. However, it presents some drawbacks such as volatility, instability and poor water solubility [21]. To overcome these constraints, in the last years, various materials such as, polylactic acid, polysaccharides, proteins and inorganic carriers have been tested for limonene encapsulation [21]. The combination of a limonene core with a lignin-based shell would result in a fully biobased and environmentally friendly delivery system, which could benefit from a synergistic combination of all the beneficial characteristics of both substances.

Additionally, this work focuses on understanding the main factors governing the characteristics of the LMCs and, on these bases, aims at providing suitable and easily scalable solutions to tailor the release behavior of the produced LMCs. The process parameters, with particular attention to lignosulfonate:limonene ratio, were screened with the aim of extrapolating structure-property relationships useful to tune the LMCs features. The obtained LMCs were characterized in terms of their size and properties of the capsule-forming softwood lignosulfonate (SLS). Lastly, with the purpose of achieving better control over the pH-responsiveness of LMCs, two approaches were followed: (i) lignosulfonate was fractionated by means of a solvent-based technique to achieve more homogeneous fractions subsequently employed for the preparation of LMCs and (ii) a specific biobased additive was designed and synthesized to be subsequently added to the formulation for LMCs production.

## 2. Experimental

### 2.1. Materials

Softwood lignosulfonate (SLS, C: 39.3%, H: 4.3%, N: <0.1%, S: 5.7%, aliphatic OH: 2.33 mmol/g, phenolic OH: 1.57 mmol/g, carboxylic groups: 0.95 mmol/g) was used for the experiments. The ^31^P spectrum of SLS is reported in Figure S1.

(*R*)-(+)-limonene (97%), thionyl chloride (99.5%), triethylamine (Et_3_N, HPLC grade) and dimethyl sulfoxide (ChromAR, HPLC grade) were purchased by Alfa Aesar (Kandel, Germany), Acros Organics, VWR (Radnor, PA, USA) and Macron (Pittsburgh, Pennsylvania, USA), respectively. Sebacic acid (99%), veratryl alcohol (96%), sodium carbonate (anhydrous, highly pure), sodium bicarbonate (99.7%), mesitylene (≥98%), hexane (≥99%), methanol (≥99.8%), tetrahydrofuran (≥99.8%) and dichloromethane (≥99.9%) were purchased from Sigma Aldrich (St. Louis, MO, USA). All reagents and solvents were used as received unless otherwise stated. MilliQ water was employed.

### 2.2. Synthesis of SLS Microcapsules (LMCs)

**Figure 1:**
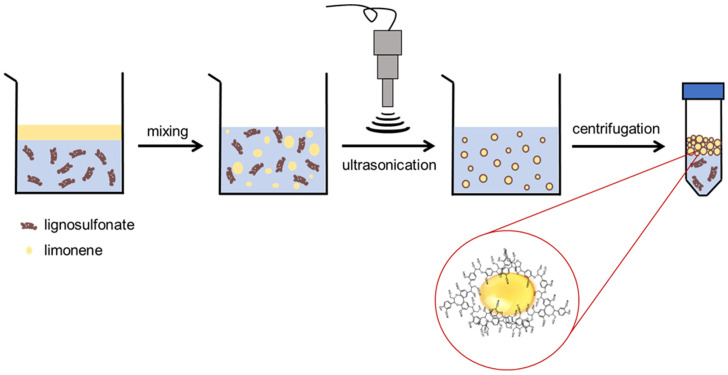
Cartoon showing the steps leading to the formation of LMCs.

A 2 wt% aqueous solution of SLS and limonene were mixed in 4:1, 3:1, 2:1, 1:1 and 0.8:1 *v*/*v* ratios (3 mL of limonene were used for all the experiments) and left under vigorous stirring for 2 min to promote the formation of an emulsion, which was sonicated at 40% amplitude for 10 min using a Branson Digital Sonifier (Model 450 L, Ultrasonic Corporation, Danbury, CT, USA) equipped with a 20 KHz Branson probe ending in a horn tip (max amplitude 160 W). Lastly, LMCs were collected by centrifugation (20 min at 9000 rpm by means of a Sartorius Centrisart G-16, Göttingen, Germany) and subsequent removal of the aqueous phase (Figure 1).

For the generation of the LMCs containing the additive, a 10 wt% diveratryl sebacate (DS) with respect to limonene was added to the limonene before the emulsification step.

### 2.3. Characterization of LMCs

Laser diffraction analysis (LDA) was employed to carry out dimensional characterization of LMCs. A Malvern Mastersizer 3000 (Malvern, UK) equipped with a Hydro EV wet dispersion unit, including an in-line sonication probe for agglomerate dispersion, was used. Analyses of suspended LMCs in MilliQ water were run at 25 °C. Spherical particles, fine powder mode sensitivity and narrow mode particle distribution were considered for the software elaboration of the results. At least 10 measurements were recorded for each sample.

For optical microscopy measurements, LMCs were diluted in distilled water in order to prepare 1 ppm dispersions, subsequently transferred on a microscope glass slide which was covered with a coverslip prior to analysis. Fluorescence microscopy studies were performed using Coumarin 6 (Sigma Aldrich, St. Louis, MO, USA). A 0.5 mg/mL solution of Coumarin 6 in limonene was prepared. LMCs were subsequently synthesized as described above. All images were obtained using an optical microscope Nikon Eclipse Ti2 (Amsterdam, The Netherlands).

### 2.4. SLS Fractionation

Fractionation was carried out by suspending 5 g of SLS powder into 250 mL of methanol. The mixture was left under stirring at 25 °C for 16 h. The soluble fraction (MSLS) was separated from the insoluble one (MILS) by centrifugation (9000 rpm for 20 min) and the solvent was removed by rotary evaporation (carried out at 40 °C). Both fractions were dried in oven at 40 °C to constant weight, yielding 59% of MSLS and 41% of MILS.

### 2.5. Synthesis of Diveratryl Sebacate (DS)

**Figure 2:**
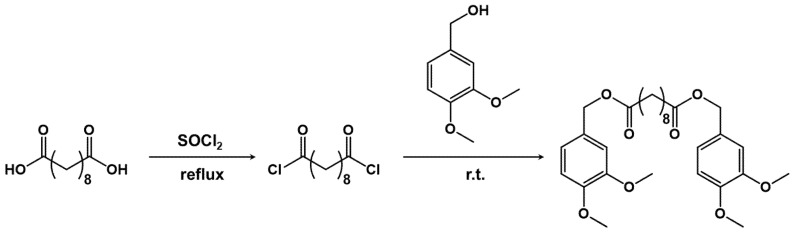
Synthetic path for diveratryl sebacate.

Diveratryl sebacate was synthesized following a two-step modified literature protocol (Figure 2) [22].

In the first step, 80 mmol of sebacic acid were placed in a three-neck round-bottom flask, equipped with a rubber septum, a condenser with a calcium chloride/sodium carbonate trap and a dropping funnel. Afterwards, 320 mmol of thionyl chloride were added dropwise. The mixture was heated to 75 °C and kept at this temperature for 2 h. Afterwards, the excess of thionyl chloride was removed under vacuum. In the second step, 40 mL of dichloromethane were added to the sebacoyl chloride prepared in the first step and the solution was mixed dropwise with a dichloromethane solution of veratryl alcohol (137 mmol) and triethylamine (194 mmol) contained in a two-neck round-bottom flask equipped with a condenser with a calcium chloride/sodium carbonate trap and a dropping funnel. The reaction was kept at room temperature for 24 h under stirring. Afterwards, the product was washed with 250 mL of a saturated solution of sodium bicarbonate (3×), the organic phase was separated and dried with sodium sulphate. Finally, the solvent was removed by rotary evaporation (carried out at 30 °C) and the obtained yellowish solid was dried under vacuum to remove Et_3_N and HCl traces (final yield > 99%). The structure was confirmed by ^1^H NMR (Figure S2).

### 2.6. Recovery and Characterization of Capsule-Forming and Unreacted SLS

To collect unreacted SLS from the aqueous phase, the solvent was removed by rotary evaporation (carried out at 40 °C) followed by drying in vacuum oven at 40 °C to constant weight. On the other hand, the recovery of capsule-forming SLS was performed as follows. LMCs were destroyed by adding THF and, after vigorous mixing, the sample was centrifuged at 9000 rpm for 10 min. After the removal of the solvent, the procedure (THF addition, mixing and subsequent centrifugation) was repeated once (no limonene was detected in the THF solution after the second extraction). Finally, the recovered SLS was dried in oven at 40 °C to constant weight prior to further analysis.

The yield of capsule-forming SLS and unreacted SLS were determined by gravimetry.

Gel permeation chromatography (GPC) was operated on a Shimadzu HPLC system (Kyoto, Japan) by employing a PLgel 5 µm MiniMIX-C column (250 × 4.6 mm). HPLC-grade DMSO containing 0.1% lithium chloride was used as eluent (0.2 mL/min, 70 °C). Standard calibration was performed with polystyrene sulfonate standards (Sigma Aldrich, 4.3–2600 kDa) and lignin model compounds (330–640 Da). A total of 8 standards were analyzed and interpolated with a third-order polynomial regression (R^2^ = 0.99). The samples were dissolved in HPLC-grade DMSO to reach a final concentration of 2 mg/mL and filtered through a 0.2 μm PTFE filter prior to injection.

^1^H-NMR and ^13^C-NMR spectra were collected on a Bruker (Billerica, MA, USA) Magnet System spectrometer 400 Ascend (^1^H: 400 MHz, 16 scans; ^13^C: 100.6 MHz, 4096 scans). Analyses were performed in CD_3_Cl. The chemical shifts (δ) in the ^1^H and ^13^C-NMR spectra were reported in parts per million (ppm).

Elemental analyses were performed using an Elementar UNICUBE instrument (Langenselbold, Germany). Accurately weighted and dried samples (1.8 ± 0.2 mg) were put onto a tin foil with the same amount of catalyst to be folded and tightly pressed prior to the analysis.

### 2.7. Evaluation of the Amount of Encapsulated Limonene

To evaluate the percentage of limonene not incapsulated in the LMCs, freshly prepared batches were treated as follows. Water and hexane in a 1:5 ratio (typically 2 mL and 10 mL, respectively) were added to the vial containing a full batch of LMCs (prepared as reported in Section 2.2) and vigorously shaken. The mixture was centrifuged at 9000 rpm for 10 min, the uppermost organic phase was removed and 100 µL of internal standard solution (IS, mesitylene in hexane at 0.1 g/mL) were added prior to GC-MS analysis.

Analyses were performed using a Shimadzu GCMS-QP2010 Ultra system (gas chromatograph GC2010 Plus) at 70 eV ionization energy. A Supelco fused-silica capillary column SLB-5 ms (30 m long, 0.25 mm thick, 0.25 μm diameter) was used as stationary phase, He (UHP grade) as mobile phase. An initial temperature of 50 °C with a ramp rate of 10 °C/min to reach a final temperature of 200 °C was used. Split injection (90:10 split ratio, injection volume: 1 μL) was chosen. System control and analyses were carried out by Shimadzu analysis software package Lab Solutions–GC-MS Solution Version 2.61. Each experiment was carried out in triplicate. The amount of encapsulated limonene was then determined by difference with the quantity initially added to the formulation.

### 2.8. Shelf Life Stability Test

To evaluate the long-term stability of LMCs, freshly prepared batches were stored in the dark at 25 °C in closed vials. At predetermined time intervals (0, 3 and 6 weeks), sacrificial specimens (each sample being constituted of a whole batch of LMCs) were treated as described above to determine the amount of released limonene.

### 2.9. pH Stability Test

LMCs stability at different pH (namely, 4, 7 and 12) and time intervals (24 and 168 h) was evaluated by incubating the LMCs at 25 °C under gentle and constant agitation. For pH 4 and pH 12, 10 mL of a CH_3_COOH/CH_3_COONa (0.1 M) buffer solution and 10 mL of a Na_2_HPO_4_/NaOH (0.1 M) buffer solution were respectively employed. MilliQ water (10 mL) was used for tests at pH 7. To evaluate the amount of released limonene at the end of the incubation period, the dispersion was centrifuged at 9000 rpm for 10 min and the LMCs were treated as above reported.

## 3. Results and Discussion

### 3.1. Generation of LMCs from Pristine SLS

The peculiar characteristics of lignosulfonate, which possess an amphiphilic backbone characterized by a high concentration of hydroxyl and sulfonate groups linked to hydrophobic aromatic rings, make it an ideal candidate for the generation of microcapsules from oil in water emulsions [11]. The oily phase, in this case, limonene, constitutes the core, while the lignin molecules, dissolved in the aqueous phase, would form the capsules’ shell. The process made possible by the self-assembly ability of this biopolymer is promoted by the application of ultrasound power. This permits, due to cavitation, reaching localized hotspots of extreme pressures and temperatures, resulting in the formation of an emulsion because of the intense mixing of the two phases [23]. The hydrophobic interactions with the oil droplets would then cause a reorientation of the lignosulfonate chains, ultimately generating the desired water-dispersible core-shell microstructures, i.e., the LMCs.

**Figure 3:**
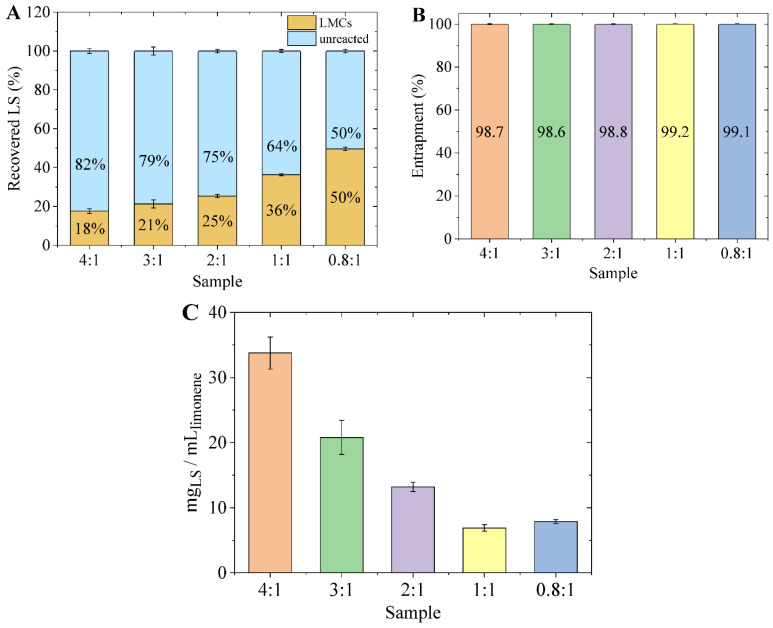
(**A**) Percentage of capsule forming SLS and of unreacted SLS; (**B**) entrapment of limonene in LMCs; (**C**) mass of SLS used per volume of limonene.

In this work, various SLS:limonene ratios were selected and tested to evaluate the effect on both the amount of lignin used for LMCs formation and on the properties of the obtained capsules. The recovery of the LMCs by centrifugation allowed the achievement of a well separated biphasic system where LMCs lie above the aqueous solution (Figure S3). As a certain concentration of SLS could be detected in the aqueous phase after the preparation of the LMCs, suggesting that not all the lignosulfonate in the starting formulation contributed to form the shell of the capsules, the amount of capsule-forming and unreacted SLS were quantified. The results, collected in Figure 3A, show that, with respect to the feed, the percentage of SLS that constitutes the LMCs increases with the decrease in the SLS:limonene ratio, reaching 50% in the case of the 0.8:1 sample.

On the other hand, Figure 3B highlights that in all cases nearly 100% of the limonene was encapsulated in the LMCs. Because of the treatment with hexane, the free limonene dispersed within the capsule layer was extracted and quantified. Only a small percentage, in all cases below 1.5% percent, was measured. On the contrary, no limonene was detected in the underneath aqueous phase. This result can be ascribed to the low water solubility (13.8 mg/L at 25 °C) and density of limonene (840 g/cm^3^) [24]. Non-encapsulated limonene would in fact lie within the capsules’ layer or above (Figure S4). Successful encapsulation of limonene was also confirmed by fluorescence microscopy analysis (Figure S5). Indeed, LMCs prepared using limonene loaded with Coumarin 6 clearly evidence a uniform and even distribution of the dye within the whole volume of the capsules.

Based on these data, the ratio between the mass of SLS employed to generate the LMCs and the volume of limonene entrapped in the capsules’ core was calculated (Figure 3C). It can be observed that, for smaller SLS to limonene ratios, LMCs were formed utilizing less mass of SLS per mL of limonene. In other words, the greater the amount of SLS available in the initial formulation, the greater the quantity of unreacted SLS but also the higher the amount of SLS used to generate the capsules’ shell.

**Figure 4:**
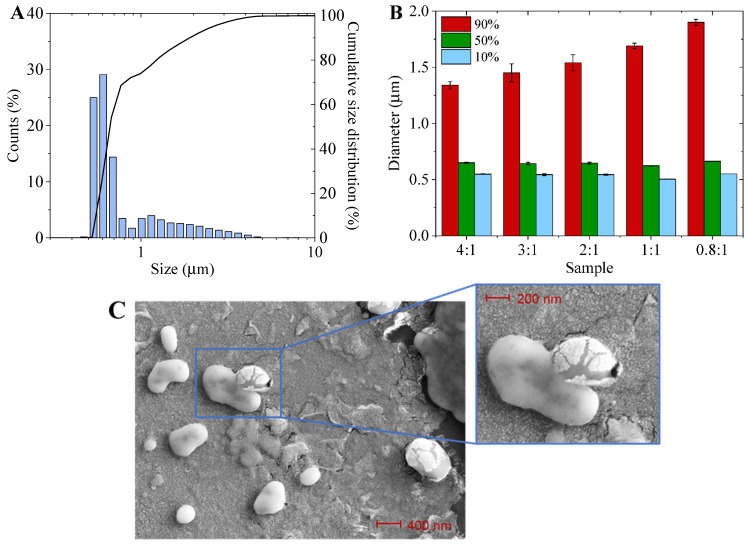
(**A**) size distribution of the generated LMCs for the sample 0.8:1; (**B**) percentage of LMCs falling below a specific diameter value (90%, red bars, 50% green bars, 10% light blue bars); (**C**) FE-SEM micrographs of LMCs prepared with a 0.8:1 SLS to limonene ratio.

The size of the LMCs may play an important role in this respect. Therefore, the dimensions of the produced LMCs were investigated by LDA analysis. Figure 4A displays the numerical size distribution for the 0.8:1 sample (Figure S6A collects the whole set of samples), while Figure 4B reports the percentages (90, 50 and 10%) of LMCs falling below a certain diameter value. Figure 4C collects SEM micrographs of LMCs. The leakage of limonene upon the breaking of the capsules’ shell is clearly evidenced.

In terms of size distribution, no significant differences can be observed among the samples for the smaller LMCs, since, in all cases, 50% of LMCs display a diameter below 0.66 µm. On the other hand, the upper diameter limit for 90% of the formed capsules increases by decreasing the SLS:limonene ratio.

Given that (i) smaller SLS:limonene ratios resulted in bigger particles (i.e., displaying a lower surface-area-to-volume ratio), (ii) the same amount of limonene was encapsulated in the LMCs irrespectively of the SLS:limonene ratio and (iii) the amount of SLS employed to form the LMCs shell per mL of limonene decreased with the decrease of SLS:limonene ratio (Figure 3C), it can be inferred that the shell thickness of the LMCs does not significantly vary among the samples. As a matter of fact, LMCs synthesized from higher SLS:limonene ratios are smaller, meaning that more SLS is required for the overall shell formation since the surface-area-to-volume ratio is relatively higher. On the other hand, by decreasing the SLS:limonene ratio, LMCs are bigger, hence, less SLS is involved in the shell formation. Sample 0.8:1 does not follow this trend as, in light of an increased capsule diameter, the amount of SLS per volume of limonene is slightly higher than the 1:1 sample, indicating that thicker capsules are formed.

### 3.2. Factors Governing the Characteristics of LMCs

Due to the well-known heterogeneous nature of lignosulfonate, the characteristics of the capsule-forming and unreacted SLS were studied in terms of molecular weight and compositions, with a specific focus on the content of sulfonate groups to evaluate any possible differences between the two fractions.

Table 1 summarizes the number average molecular weight (M_n_) and the polydispersity index (PDI) values, while Figure S7 displays the GPC elugrams of SLS recovered in the leftover aqueous phase and after the destruction of LMCs as compared with the starting lignosulfonate. From the GPC data, it can be observed that during LMCs synthesis the higher molecular weight fraction forms the capsule shell, while the lower molecular weight one is left in the aqueous phase.

**Figure 5:**
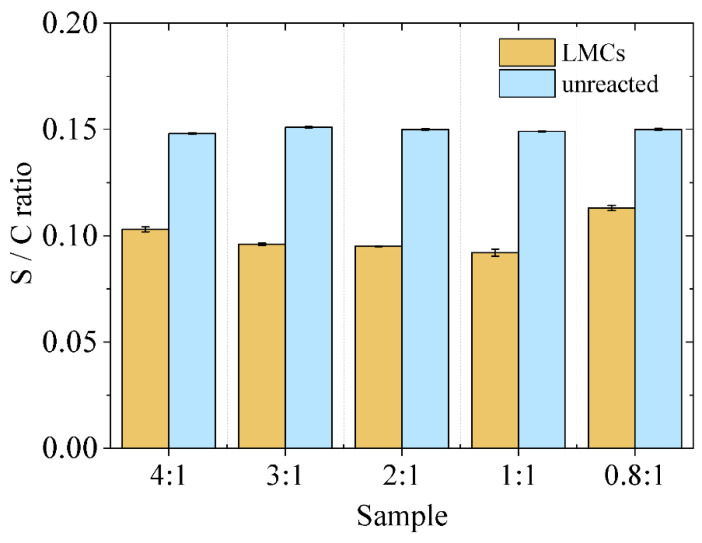
Sulfur to carbon ratio of capsule-forming and unreacted SLS.

Elemental analysis shed further light on the properties of the SLS generating the shell of the capsules. As a matter of fact, the measurements highlighted that LMCs shells are made of a lignosulfonate fraction containing a lower amount of sulfur (lower S/C ratio) with respect to the SLS collected in the aqueous phase after the reaction (Figure 5).

It should be pointed out that a higher content of sulfur is associated with a greater concentration of sulfonate groups within the SLS chains, which increases their water solubility.

The results thus demonstrate that the characteristics of the lignosulfonate play an important role in the process of LMCs assembling. Specifically, higher molecular weight allows for more extended π–π interactions and lower water solubility is preferred since the lignin molecules located at the water–oil interface are those involved in the shell formation.

Interestingly, the 0.8:1 sample displays both a significant lowering of the molecular weight and an enhanced S/C ratio for the capsule-forming SLS with respect the other samples, implying that by selecting a lower SLS:limonene ratio—i.e., less available SLS with respect to the limonene volume—LMCs are formed anyway, even if with “lower quality” lignin.

### 3.3. Modulation of the LMCs Features

**Figure 6:**
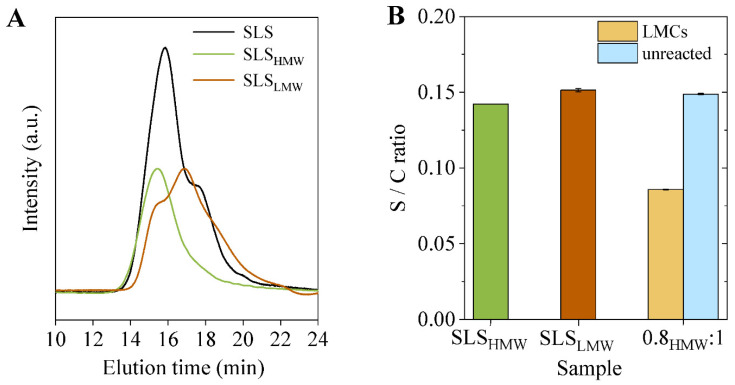
(**A**) GPC elugrams of fractionated SLS (SLS_HMW_ and SLS_LMW_) as compared to pristine SLS; (**B**) S/C ratio for fractionated SLS and for capsule-forming and unreacted lignin in LMCs produced from SLS_HMW_.

Subsequently, SLS was fractionated by a solvent-based protocol, yielding a higher molecular weight fraction (SLS_HMW_) and a lower molecular weight one (SLS_LMW_), both showing a reduced PDI with respect to the starting SLS (Table 2 and Figure 6A).

In terms of sulfonate groups content, a slightly lower S/C ratio was measured for LS_HMW_ in comparison to SLS_LMW_ (Figure 6B), suggesting that the –SO_3_^−^ functionalities are quite evenly concentrated on both higher and lower molecular weight chains.

Based on the above-reported results achieved with non-fractionated SLS, SLS_HMW_ was chosen to prepare capsules using a 0.8:1 SLS:limonene ratio (0.8_HMW_:1) and the characteristics of the LMCs were compared to those of the 0.8:1 sample.

**Figure 7:**
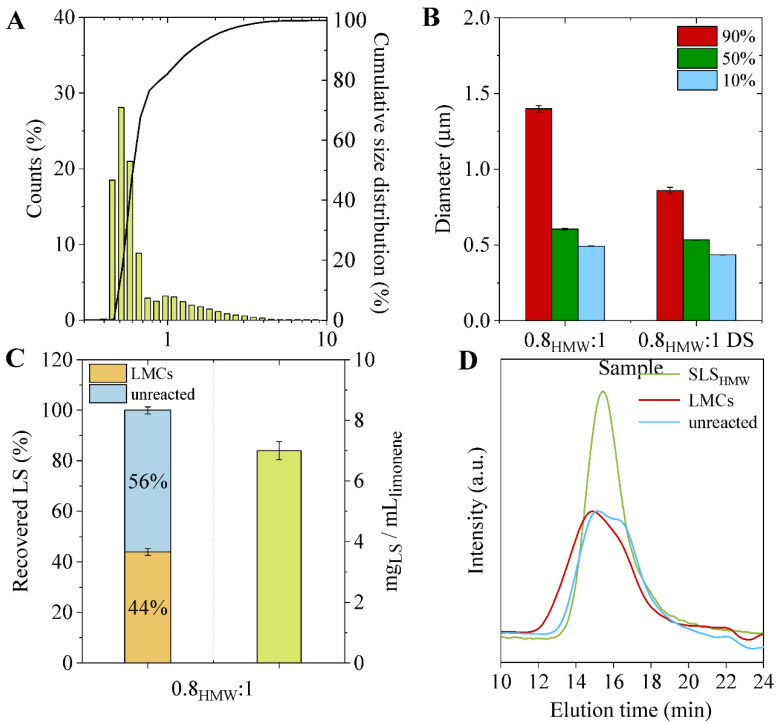
(**A**) LMCs numerical distribution of LMCs prepared from SLS_HMW_ (sample 0.8_HMW_:1); (**B**) percentage of LMCs falling below a specific diameter (90%, red bars, 50% green bars, 10% light blue bars); (**C**) percentage of capsule-forming and of unreacted SLS_HMW_ (left column) and mass of SLS_HMW_ used per volume of limonene (right column); (**D**) GPC elugrams of capsule-forming and unreacted SLS_HMW_ as compared to the feed.

In terms of the capsules’ size, although a similar distribution as compared to the samples obtained with non-fractionated SLS was measured (Figure 7A and Figure S6B), the diameter within which 90% of the capsules fall is well below that of 0.8:1 sample (1.4 vs. 1.9 µm) (Figure 7B), indicating that smaller capsules have formed. Furthermore, also in this case, not all the lignosulfonate was utilized to generate LMCs, since a significant percentage of unreacted SLS_HMW_ remained in the aqueous phase (Figure 7C). Once again, the capsule-forming SLS_HMW_ shows a higher molecular weight than the unreacted lignosulfonate (Figure 7D). However, since the molecular weight of the starting SLS_HMW_ is higher and less polydispersed than the pristine SLS, in this case, the recorded differences are less pronounced (Table 2). Last but not least, an elemental analysis confirms that lignosulfonate chains containing a lower concentration of sulfonate groups are involved in the formation of the capsules: similar S/C ratios were recorded for the capsule-forming and unreacted SLS_HMW_ with respect to the LMCs samples produced starting from pristine SLS (Figure 6B).

Capsules made from SLS_HMW_ are smaller than those prepared with unfractionated SLS using the same lignosulfonate to limonene ratio (i.e., 0:8:1), thus they exhibit a higher surface-area-to-volume ratio. Because in both samples the percentage of limonene encapsulated (99% of entrapment for the 0.8_HMW_:1 sample) and the mass of lignosulfonate per mL of limonene are similar (Figure 3C and Figure 7C), while the size of LMCs is reduced, it can be hypothesized that the capsules prepared with the SLS_HMW_ present a thinner shell than those made from unfractionated SLS.

To further modulate the functional properties of the LMCs, an ad hoc designed additive, based on green starting materials, was synthesized and introduced in the formulation. Specifically, due to its peculiar chemical structure, which contains two aromatic rings, diveratryl sebacate (DS) is expected to interact with the lignosulfonate molecules by π–π forces, while the presence of a long aliphatic chain increases the flexibility of the system, favoring a smooth incorporation in the LMCs shell. LMCs were produced starting from SLS_HMW_ and limonene in 0.8:1 ratio, adding 10 wt% of DS (0.8_HMW_:1 DS sample), yielding capsules with the smallest diameter among the samples under investigation (Figure 7B).

### 3.4. Shelf Life and pH Responsive Behavior of LMCs

In view of potential applications of the generated LMCs as industrial or biomedical carriers of fragrances, drugs, or other active compounds, both the long-term shelf stability and the response to external stimuli must be investigated. To this purpose, the as-prepared capsules were (i) stored at 25 °C for up to six weeks and (ii) incubated at 25 °C under different pH values, namely, 4, 7 and 12, to evaluate the leakage of limonene.

**Figure 8:**
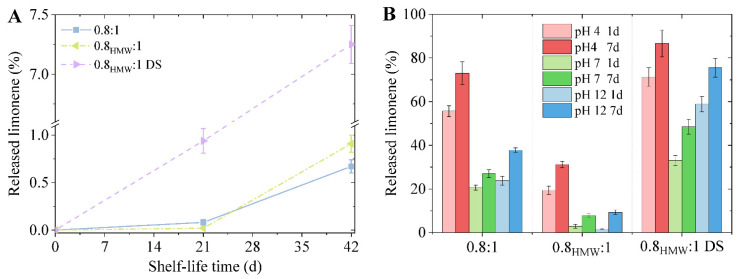
(**A**) shelf-life stability; (**B**) pH stability of LMCs samples.

The analyses carried out on a time scale of six weeks evidenced high shelf life, as less than 1% of the entrapped limonene was released over this time frame in the case of 0.8:1 and 0.8_HMW_:1 samples (Figure 8A), confirming the general robustness of the lignosulfonate-based LMCs [20]. Nevertheless, LMCs prepared in the presence of DS displayed a slightly lower stability, because a limonene leakage of about 7.25% was reached.

As to the pH response, previous studies reported more pronounced capsule instability in acidic pH rather than under alkaline conditions [20]. The disassembly phenomenon is mainly governed by electrostatic interactions. Specifically, in acidic conditions ion-dipole interactions (i.e., cation–π) and, for pH values above 2, the deprotonation of the sulfonate groups, leading to repulsive forces, trigger the capsules unfolding. On the other hand, notwithstanding the additional dissociation of phenolic OH (p*K_a_* ≈ 10), under alkaline conditions the presence of increasing amounts of hydroxide anions OH^−^ favors ionic interactions counteracting the cation–π forces responsible for capsules’ disassembly. The higher the pH, the better the LMCs stability.

This behavior was here confirmed, as in all cases, the amount of released limonene at the end of the incubation period is higher at pH 4 than under neutral or alkaline conditions (Figure 8B). Additionally, as expected, the longer the incubation time, the higher the amount of limonene detected outside the capsules. LMCs synthesized with a 0.8:1 ratio from pristine SLS resulted in being less stable than LMCs made from LS_HMW_, the latter displaying an outstanding stability, especially at pH ≥ 7.

This finding can be explained based on different factors. As a matter of fact, the release of hydrophobic molecules confined in biodegradable polymers depends on several factors, such as solvent penetration into the polymeric matrix, polymer degradation and drug diffusion [25].

Due to the lower shell thickness of the 0.8_HMW_:1 sample, which should lead to an increased in/out diffusion in both the solvent and limonene, the major role for the release of limonene is played by the “degradation” of the shell and more specifically by the chemical structure of the lignosulfonate chains that form the LMCs. The 0.8:1 sample displays a higher release of the active because of the lower molecular weight and the greater concentration of sulfonate groups which enhance the water solubility of SLS molecules.

Last but not least, the addition of DS permitted to significantly modify the kinetic of limonene release, specifically resulting in an enhanced capsule instability also under neutral and alkaline conditions due to the following reasons: (i) the two ester bonds can be cleaved under acidic or alkaline pH, hence leading to the disassembly of the LMCs; (ii) the long aliphatic chain may interfere with lignin π–π stacking, hampering the formation of a dense and defect-free shell; and (iii) the capsules are much smaller than the other samples, showing a higher surface area to volume ratio.

## 4. Conclusions

Limonene-core lignosulfonate-shell microcapsules were successfully generated by an ultrasound-assisted strategy exploiting the self-assembly ability of the lignosulfonate molecules. The adopted technique is sustainable, since water is indeed the only used solvent, simple and easily scalable. The obtained LMCs are capable of encapsulating nearly 100% of the active compound and, in terms of numerical distribution, 90% of the capsules display a diameter below 2 μm. By screening the reaction parameters, with a specific focus on the SLS to limonene ratio, it was possible to identify the key physicochemical parameters for the capsule formation, i.e., lignosulfonate molecular weight and content of sulfonate groups. On these bases, two different approaches were implemented to modulate the pH-triggered release of the entrapped compound. On the one hand, lignosulfonate was fractionated into a high molecular weight fraction and a low molecular weight one. The high molecular weight fraction was afterwards employed to produce LMCs. The latter, because of the increased molecular weight and narrower PDI, coupled with a lower content of sulfonate functionalities, expressed an excellent stability both under acidic and alkaline pH conditions, with a release of limonene below 32% after 7 days of incubation at pH 4. On the other hand, the introduction of diveratryl sebacate, an ad hoc prepared biobased additive, to the LMCs formulation allowed for much faster release kinetics, reaching values above 70% after 24 h of storage both at pH 4 and pH 12. This is due to the specific structure of diveratryl sebacate that, due to the presence of aromatic rings, is capable of interacting with the SLS chains, being incorporated in the capsule shell. The two hydrolysable ester bonds promote the capsule disassembly upon pH changes.

The results here reported represent a highly efficient, yet very facile way to tailor the pH-responsiveness of LMCs, without the need for any chemical modifications of the starting lignosulfonate. They thus foster new opportunities both in terms of the valorization of a largely available waste biomass and of exploitation of environmentally friendly and biocompatible carriers for the targeted and controlled delivery of active substances in biomedicine or other widespread consumer goods.

## Figures and Tables

**Table 1:** The number average molecular weight distribution (M_n_) and polydispersity index (PDI) of capsule-forming and unreacted SLS.

Sample	M_n_		M_w_/M_n_	
	LMCs	Unreacted	LMCs	Unreacted
SLS	3000		6.3	
4:1	11,400	2500	4.5	4.7
3:1	11,600	2700	4.8	4.8
2:1	15,200	2600	4.7	4.9
1:1	12,800	2300	4.6	4.6
0.8:1	8800	2200	4.6	4.3

**Table 2:** Number average molecular weight distribution (M_n_) and polydispersity index (PDI) of pristine and fractionated SLS and of capsule-forming and unreacted SLS for the 0.8_HMW_:1 sample.

Sample	M_n_		M_w_/M_n_	
	LMCs	Unreacted	LMCs	Unreacted
SLS	3000		6.3	
SLS_HMW_	11,700		3.7	
SLS_LMW_	1500		5.5	
0.8_HMW_:1	13,500	7800	3.4	3.3

## Data Availability

The data presented in this study are available on request from the corresponding author. The data are not publicly available due to privacy restrictions.

## Supplementary Materials

The following supporting information can be downloaded at: https://www.mdpi.com/article/10.3390/ma15051857/s1, Figure S1: ^31^P-NMR spectrum of starting SLS, Figure S2: ^1^H-NMR of diveratryl sebacate, Figure S3: Photographs of LMCs after the centrifugation step, Figure S4: Photographs of LMCs (sample) after incubation at pH 4 for 24 h, Figure S5: (A) Optical and (B) fluorescence microscopy pictures of Coumarin 6-loaded LMCs prepared with a 0.8:1 SLS to limonene ratio, Figure S6: Size distribution of the generated LMCs as measured by LDA: (A) LMCs prepared from pristine SLS, (B) LMCs prepared from LS_HMW_, Figure S7: GPC elugrams of capsule-forming and unreacted SLS as compared to starting SLS.
