# Supplementary material for: Simple Strategies to Modulate the pH-Responsiveness of Lignosulfonate-Based Delivery Systems
Source: Materials (Basel). 2022 Mar 2;15(5):1857. doi: 10.3390/ma15051857 (PMC8911673; doi:10.3390/ma15051857)
Supplement: Supplementary file 1 [file materials-15-01857-s001.zip › materials-1508111-supplementary.pdf]

SUPPORTING INFO

Simple strategies to modulate the pH-responsiveness of lignosulfonate-based delivery systems

Massimo Sgarzi, Matteo Gigli, Charlotte Giuriato, Claudia Crestini

Department of Molecular Sciences and Nanosystems, Ca' Foscari University of Venice, Via Torino 155, 30172 Venezia-Mestre, Italy

Index

Figure S1. ^{31}P-NMR spectrum of starting SLS pag. 2
Figure S2. ^{1}H-NMR of diveratryl sebacate pag. 2
Figure S3. Photographs of LMCs after the centrifugation step pag. 3
Figure S4. Photographs of LMCs (sample) after incubation at pH 4 for 24 h pag. 4
Figure S5. (A) Optical and (B) fluorescence microscopy pictures of Coumarin 6-loaded LMCs prepared with a 0.8:1 SLS to limonene ratio pag. 4
Figure S6. Size distribution of the generated LMCs as measured by LDA: A) LMCs prepared from pristine SLS, B) LMCs prepared from LS_{HMW} pag. 5
Figure S7. GPC elugrams of capsule-forming and unreacted SLS as compared to starting SLS pag. 6

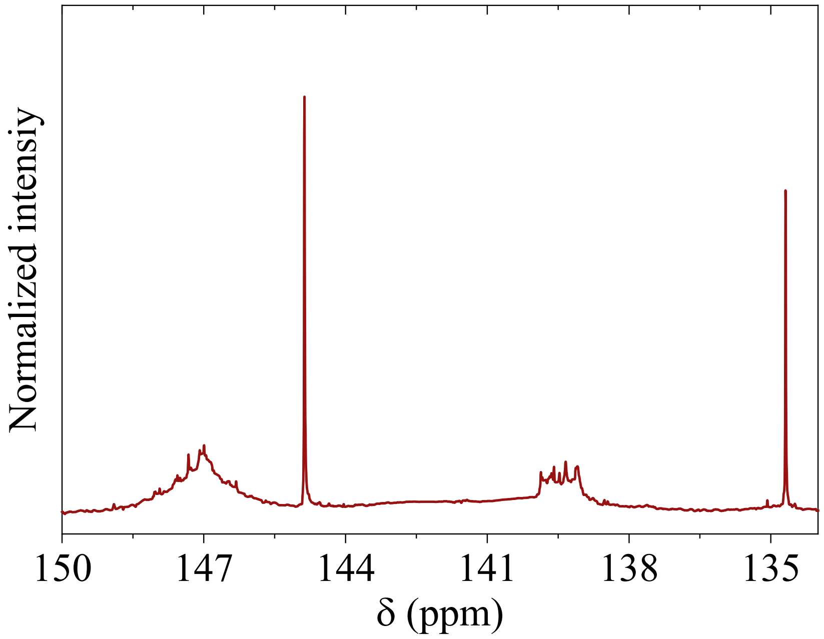

Figure S1. ^{31}P-NMR spectrum of starting SLS.

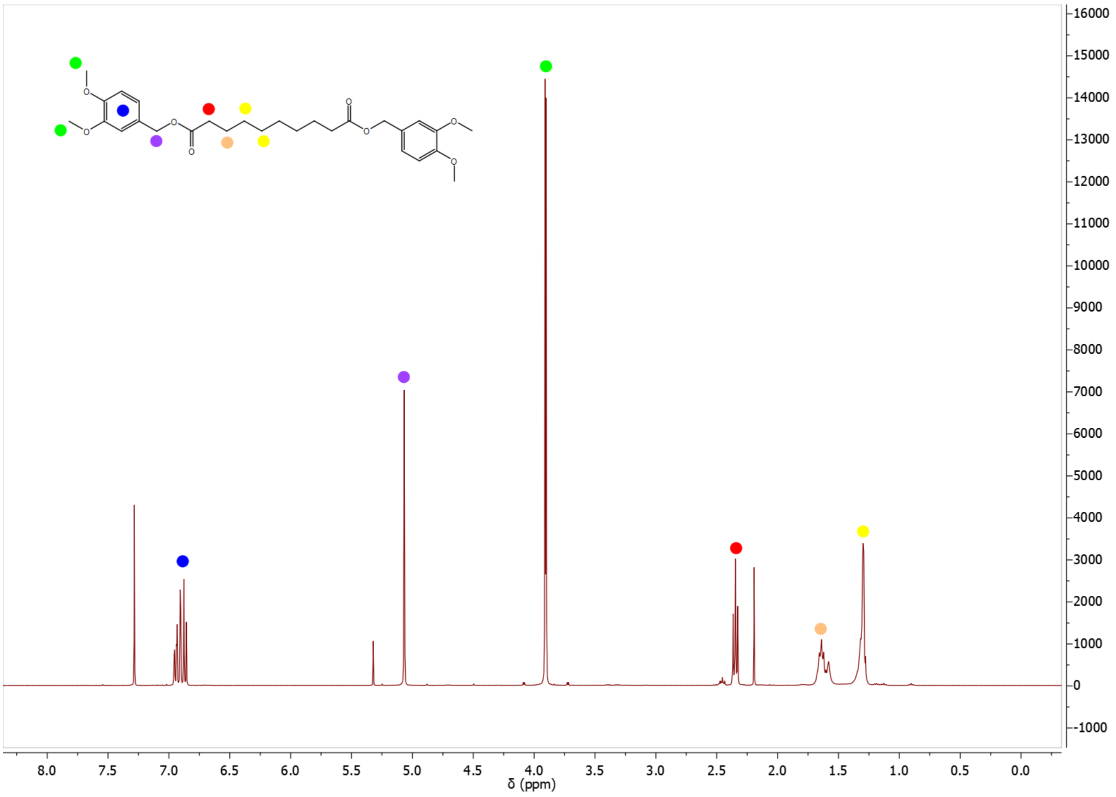

Figure S2. ^{1}H-NMR of diveratryl sebacate (400 MHz, $CDCl_3$, 298 K): δ = 6.85 (m, 6H), 5.06 (s, 4H), 3.91-3.90 (s, 12H), 2.32 (t, J = 7.5 Hz, 4H), 1.60 (m, 4H), 1.27 (m, 8H) ppm.

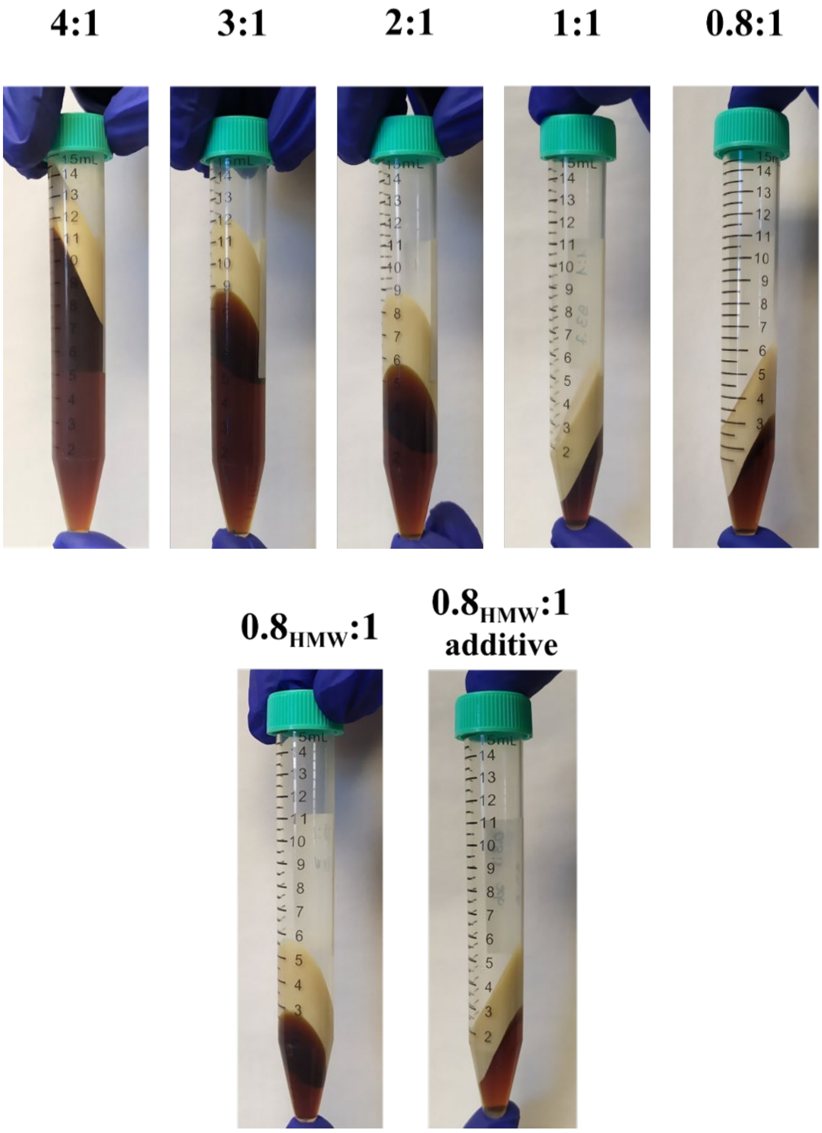

Figure S3. Photographs of LMCs after the centrifugation step.

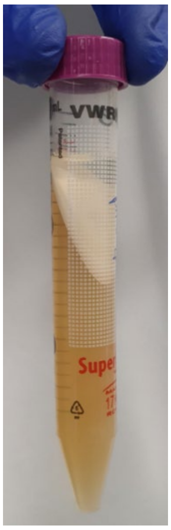

Figure S4. Photographs of LMCs (sample) after incubation at pH 4 for 24 h. The upper phase constituted of limonene released from the capsules is clearly visible above the capsule layer. The aqueous phase is colored due to the partial unfolding of the LMCs shell which liberated a certain amount of SLS molecules.

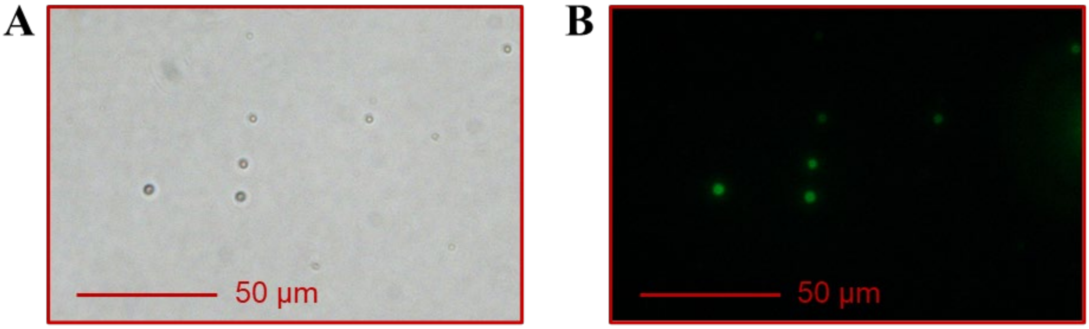

Figure S5. (A) Optical and (B) fluorescence microscopy pictures of Coumarin 6-loaded LMCs prepared with a 0.8:1 SLS to limonene ratio. Coumarin 6 is visible within the whole volume of the capsules.

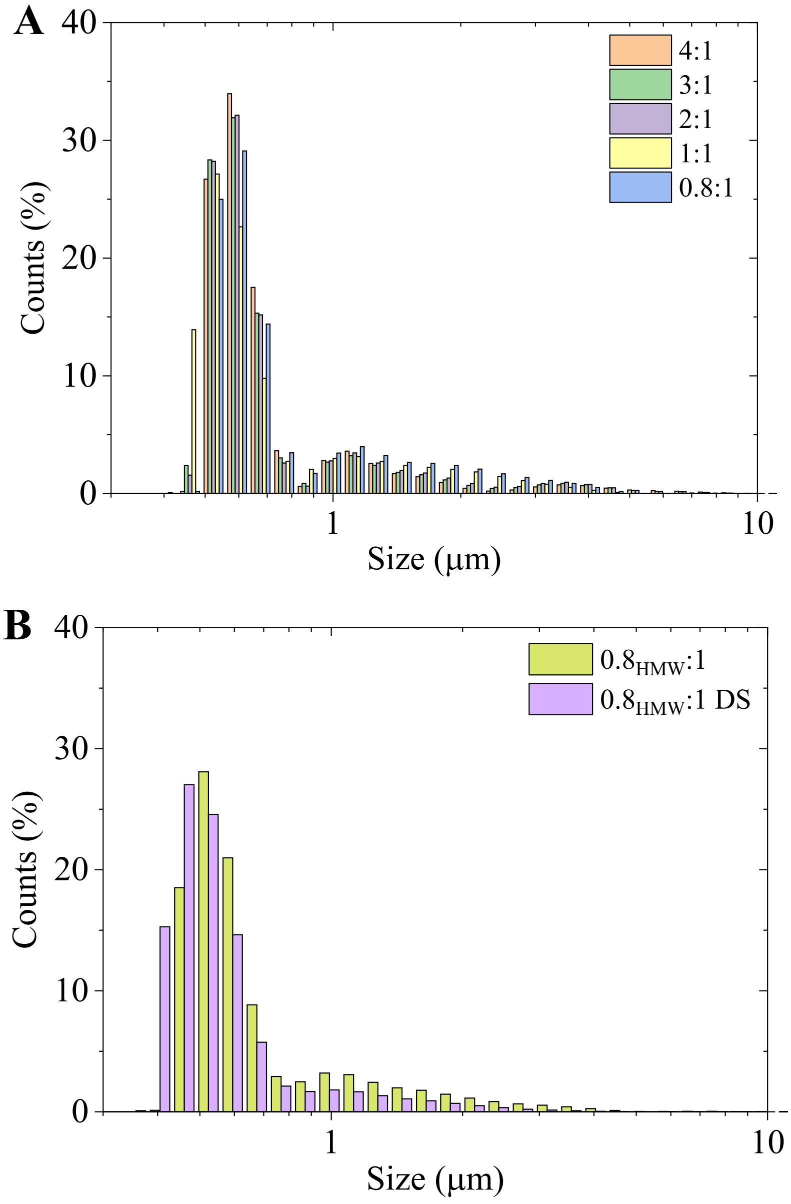

Figure S6. Size distribution of the generated LMCs as measured by LDA: A) LMCs prepared from pristine SLS, B) LMCs prepared from LS_{HMW}.

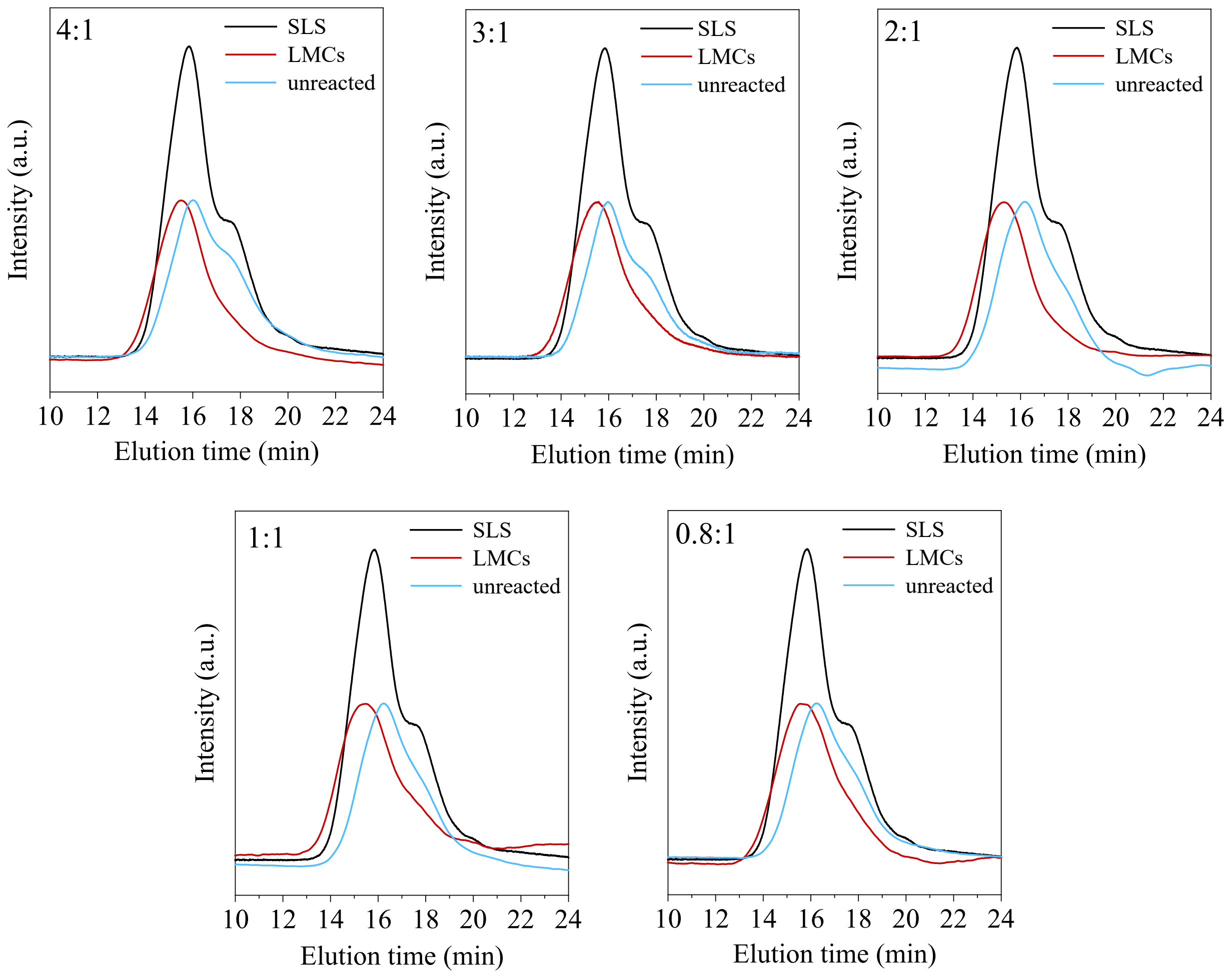

Figure S7. GPC elugrams of capsule-forming and unreacted SLS as compared to starting SLS.
